# Deregulated expression of HDAC9 in B cells promotes development of lymphoproliferative disease and lymphoma in mice

**DOI:** 10.1242/dmm.023366

**Published:** 2016-12-01

**Authors:** Veronica S. Gil, Govind Bhagat, Louise Howell, Jiyuan Zhang, Chae H. Kim, Sven Stengel, Francisco Vega, Arthur Zelent, Kevin Petrie

**Affiliations:** 1 Division of Clinical Studies, Institute of Cancer Research, London SM2 5NG, UK; 2 Department of Pathology & Cell Biology, Columbia University Medical Center, New York, NY 10032, USA; 3 Department of Pathology, Herbert Irving Comprehensive Cancer Center, Columbia University Medical Center, New York, NY 10032, USA; 4 Division of Molecular Pathology, Institute of Cancer Research, London SM2 5NG, UK; 5 Institute for Cancer Genetics, Columbia University, New York, NY 10032, USA; 6 Division of Hematopathology, Sylvester Cancer Center, University of Miami, Miami, FL 33136, USA; 7 Department of Medicine, Sylvester Comprehensive Cancer Center, University of Miami, Miami, FL 33136, USA; 8 Department of Biological and Environmental Sciences, Faculty of Natural Sciences, University of Stirling, Stirling FK9 4LA, UK

**Keywords:** HDAC9, Lymphoma, Transgenic mouse

## Abstract

Histone deacetylase 9 (HDAC9) is expressed in B cells, and its overexpression has been observed in B-lymphoproliferative disorders, including B-cell non-Hodgkin lymphoma (B-NHL). We examined HDAC9 protein expression and copy number alterations in primary B-NHL samples, identifying high HDAC9 expression among various lymphoma entities and *HDAC9* copy number gains in 50% of diffuse large B-cell lymphoma (DLBCL). To study the role of HDAC9 in lymphomagenesis, we generated a genetically engineered mouse (GEM) model that constitutively expressed an *HDAC9* transgene throughout B-cell development under the control of the immunoglobulin heavy chain (IgH) enhancer (*Eμ*). Here, we report that the *Eμ-HDAC9* GEM model develops splenic marginal zone lymphoma and lymphoproliferative disease (LPD) with progression towards aggressive DLBCL, with gene expression profiling supporting a germinal center cell origin, as is also seen in human B-NHL tumors. Analysis of *Eμ-HDAC9* tumors suggested that HDAC9 might contribute to lymphomagenesis by altering pathways involved in growth and survival, as well as modulating BCL6 activity and p53 tumor suppressor function. Epigenetic modifications play an important role in the germinal center response, and deregulation of the B-cell epigenome as a consequence of mutations and other genomic aberrations are being increasingly recognized as important steps in the pathogenesis of a variety of B-cell lymphomas. A thorough mechanistic understanding of these alterations will inform the use of targeted therapies for these malignancies. These findings strongly suggest a role for HDAC9 in B-NHL and establish a novel GEM model for the study of lymphomagenesis and, potentially, preclinical testing of therapeutic approaches based on histone deacetylase inhibitors.

## Abstract (precis)

**Summary:** This study demonstrates that aberrant expression of HDAC9 in B cells promotes development of lymphoproliferative disease and lymphoma through altering expression of genes involved in the cell cycle and survival, and modulating the activity of key B-lineage factors such as BCL6 and p53.

## INTRODUCTION

Non-Hodgkin lymphoma (NHL) are a heterogeneous group of cancers of B, T or natural-killer cells, and constitute 4-5% of all cancers (Ferlay et al., 2013; Siegel et al., 2015), with diffuse large B-cell lymphoma (DLBCL) being the most common subtype, accounting for 31% of all adult NHLs (Martelli et al., 2013). Overall, approximately five cases of NHL per 100,000 individuals are identified annually (rising to 12 per 100,000 in North America), with incidence increasing, especially in developed countries. Similarly, NHL is the 11th most common cause of cancer death worldwide, resulting in around 200,000 deaths in 2012. Despite improvements in 5-year relative survival rates to 70% over the last four decades (largely due to the use of antibodies and antibody–drug conjugates directed against cell-surface antigens), patients with relapsed or refractory disease continue to have poor outcomes (Ansell, 2015; Grover and Park, 2015). New approaches are therefore required in NHLs, and therapeutic targeting of epigenetic modifiers, including histone deacetylases (HDACs), holds great promise (Hassler et al., 2013).

HDACs catalyze deacetylation of acetylated lysine residues on histones, and are also being found to act on a growing number of non-histone proteins (Haberland et al., 2009; Yang and Seto, 2008). As such, the functional interaction networks of HDACs encompass many biological and cellular processes beyond chromatin modification and gene regulation. In humans, there are 11 canonical HDACs grouped into three major classes: class I comprises HDACs 1, 2, 3 and 8; class II comprises HDACs 4, 5, 6, 7, 9 and 10; and class IV is represented by HDAC11. HDAC9, alongside HDACs 4, 5 and 7, forms the class IIa subfamily, and these proteins are key transcriptional co-regulators in development and differentiation (Martin et al., 2009). Mutation or aberrant expression of *HDAC9* has been implicated in diverse conditions, including ischemic stroke, schizophrenia and obesity (Bellenguez et al., 2012; Chatterjee et al., 2014; Lang et al., 2012), and also as a maker of poor outcome in cancer (Milde et al., 2010; Moreno et al., 2010). *HDAC9*, which is subject to complex regulation via differential promoter usage and alternative splicing, is preferentially expressed in the lymphoid lineage within the hematopoietic system (Petrie et al., 2003). HDAC9 is highly expressed in B-lymphoproliferative disorders, including in B-cell non-Hodgkin lymphoma (B-NHL) cell lines and patient samples, suggesting that its deregulation might lead to abnormal B-cell proliferation (Petrie et al., 2003; Sun et al., 2011). These findings are supported by the recurrent amplification of the *HDAC9* locus (chr. 7p21.1) in B-NHL (Bea et al., 2005; Bentz et al., 1999, 1996; Monni et al., 1996; Rubio-Moscardo et al., 2005; Tagawa et al., 2005). Additionally, a number of HDAC inhibitors have been shown to induce cell death in B-NHL cells (Haery et al., 2015; Lemoine and Younes, 2010). Although several *in vivo* mouse models examining the biological functions of the class I and II HDACs are available (Witt et al., 2009), a role for HDAC9 or other family members in B-NHL has not been examined *in vivo*. The study of *Hdac9*^–/–^ knockout mice has, however, highlighted HDAC9 as an important factor in inhibiting the generation and function of regulatory T (T_reg_) cells (Tao et al., 2007; Yan et al., 2011).

Underlining a potential role in B-NHL, HDAC9 interacts with BCL6 (Basso et al., 2010; Miles et al., 2005; Petrie et al., 2003), a transcriptional repressor that is crucial for germinal center (GC) formation (Basso and Dalla-Favera, 2012). Transgenic mice that constitutively express BCL6 in B cells develop a lymphoproliferative syndrome that culminates with the development of B-NHL (Cattoretti et al., 2005). BCL6 directly recruits class-II HDACs through its zinc-finger domain (Lemercier et al., 2002), and its transcriptional targets in GC B cells include *TP53*, thus modulating DNA-damage-induced apoptotic responses (Phan and Dalla-Favera, 2004). Evidence for a major role for defective acetylation in the pathogenesis of B-NHL is supported by the frequent occurrence of structural alterations inactivating *CREBBP* and *EP300*, genes encoding two highly related histone acetyltransferases (HATs) and non-HATs (Pasqualucci and Dalla-Favera, 2015; Pasqualucci et al., 2011). These mutations lead to aberrant activation and deactivation, respectively, of BCL6 and p53 (Pasqualucci et al., 2011), and we hypothesized that aberrant HDAC9 expression could also interfere with p53 and/or BCL6. We therefore sought to characterize HDAC9 expression in human B-cell lymphomas and establish whether aberrant expression can drive B-cell lymphoma in a genetically engineered mouse (GEM) model. Here, we report the development of a GEM in which an *HDAC9* transgene was constitutively expressed in B cells under the control of the immunoglobulin heavy chain (*Eμ*) enhancer (*Eµ-HDAC9*). *Eµ-HDAC9* mice developed B-lymphoproliferative disorders with progression towards B-NHL. This is consistent with the hypothesis that deregulated protein acetylation plays a pathological role in B-NHL, and provides a model for preclinical evaluation of HDAC inhibitors (HDACIs).

## RESULTS

**Fig. 1.**
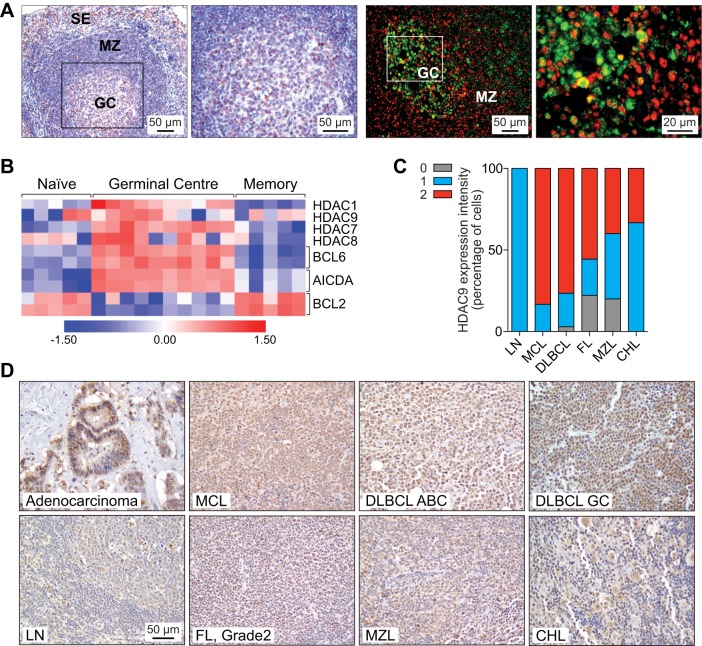
**HDAC9 is highly expressed in human B-cell lymphomas.** (A) HDAC9 expression in germinal center (GC) lymphatic nodules of normal human tonsils. Left panels, immunohistochemical staining for HDAC9 (red). Cells were nuclear counterstained with hematoxylin (blue). Right panels, immunofluorescent analysis of HDAC9 (red) and BCL6 (green) co-expression. SE, subepithelial cells; MZ, marginal zone. (B) Expression of HDAC family members in purified mature B-cell subpopulations (naive, GC, memory). Expression patterns of BCL6, AICDA and BCL2 are shown as controls. Individual columns correspond to independent samples. The color scale reflects the range in expression values after log2 transformation (0, mean expression level; red, high expression; blue, low expression). Expression data from Klein et al. (2003). (C) Average signal intensity of HDAC9 staining in the indicated samples was scored as negative (0; gray), low (1; blue) or high (2; red) relative to rectal adenocarcinoma cells expressing HDAC9. Samples containing cells expressing on average HDAC9 with equal or higher intensity were scored as 2 and samples with lower expression were scored as 1. Cells lacking expression of HDAC9 were scored as 0. (D) Representative images of HDAC9 expression. Expression of HDAC9 in rectal adenocarcinoma is shown as a positive control. DLBCL, diffuse large B-cell lymphoma; GC, germinal cell; ABC, activated B-cell; CHL, classical Hodgkin lymphoma; FL, follicular lymphoma; MCL, mantle cell lymphoma; MZL, marginal zone lymphoma; LN, reactive lymph node.

Within the immune system, a role for HDAC9 in the control of T_reg_ cell function has previously been described (Beier et al., 2012; de Zoeten et al., 2010; Parra, 2015; Tao et al., 2007), and we found that, in normal human mature B cells, *HDAC9* mRNA expression is significantly upregulated in the GC (Petrie et al., 2003) (Fig. 1A). HDAC9 protein is detected in a subset of GC cells, where it is co-expressed with BCL6 (Fig. 1A), as well as in a subset of lymphoid cells in the mantle zone and paracortex (Klein et al., 2003) (Fig. 1B). High *HDAC9* gene expression in B-lymphoproliferative disorders, including B-NHL cell lines and patient samples, has pointed to a potential role in these diseases (Petrie et al., 2003; Sun et al., 2011). In line with these findings, we detected high HDAC9 protein levels among various lymphoma entities, including DLBCL (*n*=34), marginal zone lymphoma (MZL) (*n*=5), follicular lymphoma (FL) (*n*=9), classical Hodgkin lymphoma (CHL) (*n*=3) and mantle cell lymphoma (MCL) (*n*=6). Highest levels of HDAC9 expression were observed in the most aggressive lymphomas, such as DLBCL (both GC and non-GC subtypes) and MCL (77% and 83%, respectively, *P*=1.0, Fisher's exact test). In contrast, low-grade B-cell lymphomas, as well as CHL, showed low HDAC9 expression in tumor cells when nuclear intensity was compared with that of adenocarcinoma cells as a positive control (*P*=0.004, Fisher's exact test) (Fig. 1C). In addition to high HDAC9 expression, frequent amplification of the *HDAC9* locus (chr. 7p21.1) has been observed in B-NHL (Bea et al., 2005) and, consistent with these results, we found copy number gains of *HDAC9*, including high-level amplifications, in 46.3% (25/54) of DLBCL patients (Fig. S1). A total of 46% (13/28) of samples with *HDAC9* copy number gains presented trisomy 7 (Fig. S1A), whereas 43% (12/28) of cases reported with smaller regions of amplification within the chromosome that contained the *HDAC9* gene (Fig. S1B). Here, one case displayed a specific amplification of *HDAC9* (18,409,840-18,605,177 bp) (Fig. S1C, Table S1).

**Fig. 2.**
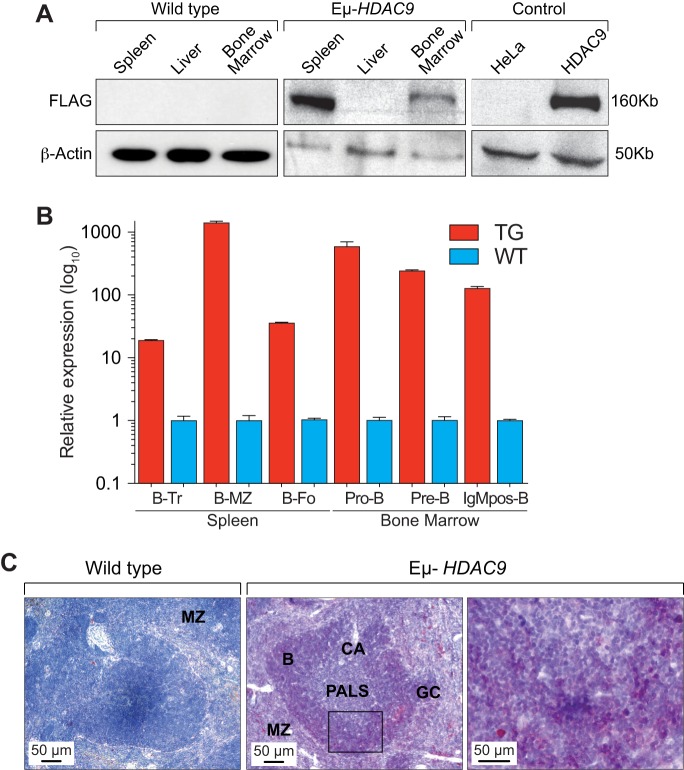
**Characterization of *Eµ-HDAC9* transgenic mice.** (A) Western blot analysis for detection of *HDAC9^TG^* expression with monoclonal anti-FLAG M2 antibody. HeLa cells, which lack expression of FLAG, and HeLa cells transfected with FLAG-tagged HDAC9 are shown as negative and positive controls, respectively. (B) *HDAC9^TG^* (TG) transcript expression in B-cell subsets. mRNA transcripts for *HDAC9* were analyzed by quantitative reverse transcription PCR (RT-qPCR) from *HDAC9^TG^* (TG, red) spleen (left) or bone marrow (right) versus controls from wild-type littermates (WT, blue). RNA from three individuals (spleen) or five individuals (bone marrow) was pooled and values are expressed as the fold change in transcript abundance (±s.d.) compared with values for WT mice. Transitional B-cell (B-Tr), *P*<0.0001; marginal zone B-cell (B-MZ), *P*<0.0001; follicular B-cell (B-Fo), *P*<0.0001; Pro B-cell (Pro-B), *P*=0.0010; Pre B-cell (Pre-B), *P*<0.0001; IgM-positive/mature B-cell (IgMpos-B), *P*<0.0001. (C) Immunohistochemical analysis of *HDAC9^TG^* expression in spleen from two *Eµ-HDAC9* mice and a wild-type littermate control. HDAC9 expression was detected with anti-FLAG M2 antibody and stained red with AEC (3-amino-9-ethylcarbazole). Cells were nuclear counterstained with hematoxylin. In *Eµ-HDAC9* spleen, expression of *HDAC9^TG^* is highest in the marginal zone and white pulp (B-cell zone). MZ, marginal zone; CA, central arteriole; PALS, periarteriolar lymphoid sheath (T-cell zone); GC, germinal center; B, primary follicles B-cell rich (B-cell zone).

Although several *in vivo* mouse models examining the biological functions of the class I and II HDACs are available (Parra, 2015), a role for HDAC9 or other class-IIa family members in B-NHL has never been examined *in vivo*. We therefore expressed a human *HDAC9* transgene (*HDAC9^TG^*) in the B-cell compartment from an early stage of B-cell development under the control of the immunoglobulin heavy chain (IgH) enhancer (*Eμ*) (Fig. S2A). We generated three independent transgenic lines (designated as 1468, 1469 and 1839) (Fig. S2B) and monitored a total of 124 mice (78 *Eµ-HDAC9* and 46 wild type) for tumor formation and overall survival. We found expression of the *HDAC9^TG^* in the bone marrow and spleen but not in the liver (Fig. 2A). We detected expression of *HDAC9^TG^* throughout all B-cell stages in the bone marrow (pro-B, pre-B and naive-B) and spleen (transitional, marginal zone and follicular), with greatest expression of *HDAC9^TG^* found in the splenic marginal zone (Fig. 2B,C). When analyzed between 6 and 12 months of age, a fraction (3/17, 18%) of *Eµ-HDAC9* mice exhibited splenomegaly (Fig. S3A,B), compared to 0/10 wild-type littermates. Histopathology and fluorescence-activated cell sorting (FACS) analysis revealed evidence of abnormal B-cell expansion in the spleen, compatible with the development of lymphoproliferative disorder (LPD) (*n*=1), and splenic MZL (SMZL) (*n*=2); no abnormalities were observed in control mice (0/10) (*P*<0.0001) (Fig. S3A-C). Analysis of immunoglobulin (Ig) gene rearrangements in these mice confirmed monoclonal expansions of B-cell populations in 2/3 young-adult *Eµ-HDAC9* mice at 8 months of age (Table 1). These results closely mirror those for a GEM model constitutively expressing BCL6 in B cells under the control of the immunoglobulin heavy chain (IgH) *Iµ* promoter (Cattoretti et al., 2005). A remarkably similar fraction of these mice (4/24, 17%) also displayed LPD at 6 months of age, representing early stages of lymphomagenesis before the evolution and onset of a B-cell neoplasm later in life (Cattoretti et al., 2005).

**Table 1.**
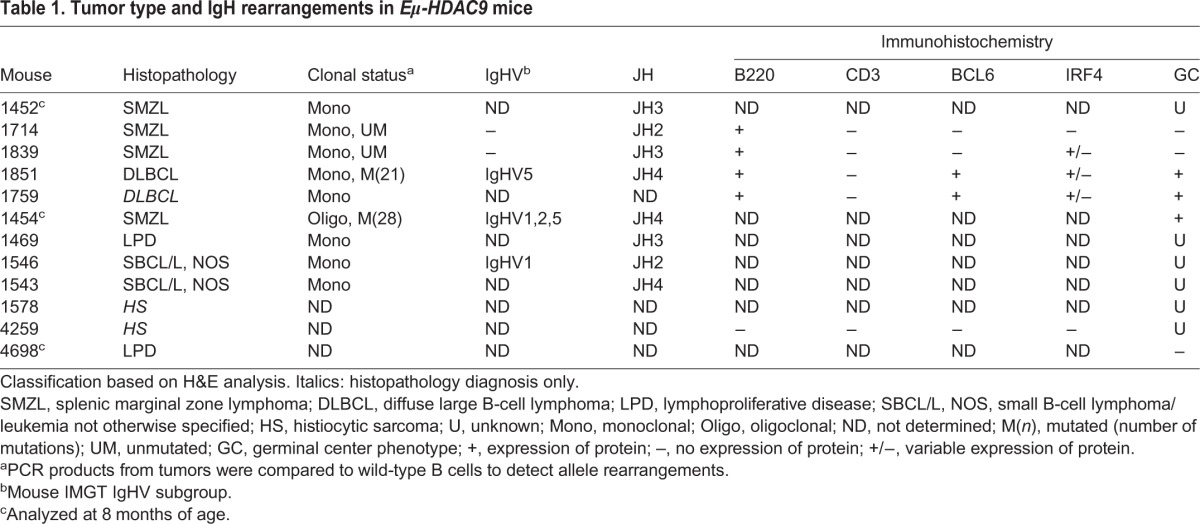
**Tumor type and IgH rearrangements in *Eμ-HDAC9* mice**

**Fig. 3.**
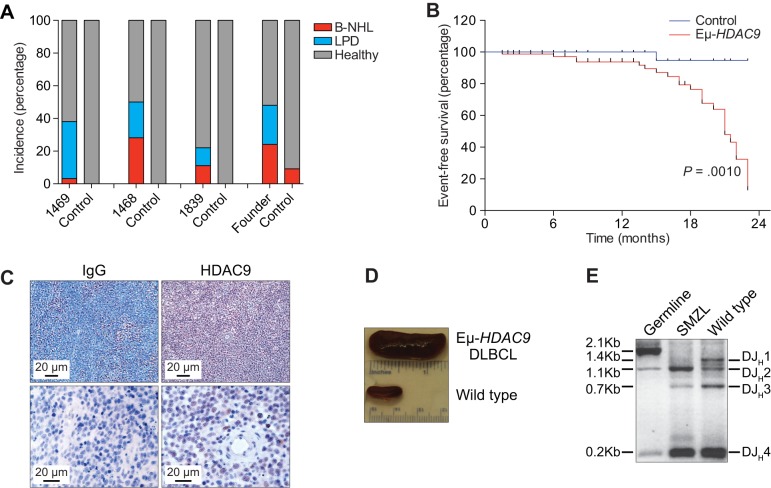
**Frequency of lymphoproliferative disease (LPD) and B-cell lymphoma (B-NHL) in *Eµ*-*HDAC9* mice.** (A) Incidence of B-NHL (red) and LPD (blue) in three *Eµ-HDAC9* lines [1469 (*n*=28), 1468 (*n*=18) and 1839 (*n*=8)] and founder mice (*n*=17) aged between 14 and 23 months as compared to wild-type (control) littermates [1469 (*n*=18), 1468 (*n*=8), 1839 (*n*=9) and founder mice (*n*=11)]. Statistical analysis was performed using; Chi-square (*χ*^2^) test on combined value for *Eμ-HDAC9* lines and founders versus wild-type controls (*P*<0.0001). (B) Kaplan–Meier plot of event-free (B-NHL) survival of transgenic versus wild-type littermate controls. *Eµ-HDAC9* (*n*=78) and control (*n*=46) mice, *P*=0.0010, log-rank (Mantel–Cox) test. (C) Immunohistochemical (IHC) analysis of HDAC9 expression (red, anti-HDAC9 antibody) in *Eµ-HDAC9* diffuse large B-cell lymphoma (DLBCL) lymph nodes. Staining for IgG (blue) was used as a control. (D) Representative *Eµ-HDAC9* spleen infiltrated by DLBCL (top) compared to wild-type littermate control spleen (bottom). (E) Analysis of rearranged IgH genes in *Eµ-HDAC9* splenic marginal zone lymphoma (SMZL). Shown is a monoclonal D-J_H_ rearrangement confirmed by sequencing (D-J_H_2, 1.1 kb). Also shown is polyclonal rearrangement of a wild-type littermate control. Tail DNA was used for germline configuration.

With age (i.e. past 14 months), *Eµ-HDAC9* mice developed a significantly higher frequency of lymphoproliferations compared to control animals, such that, by 21 months of age, 48% of *Eµ-HDAC9* mice survived compared with 95% of wild-type control mice (*P*=0.0010, Fig. 3A). Phenotypic analysis in a subset of animals revealed that approximately 40% (line 1469), 50% (line 1468) and 20% (line 1839) of *Eµ-HDAC9* mice developed B-cell lymphomas, compared to a minor fraction (5%) of wild-type littermates that exhibited evidence of B cell malignancies (*P*<0.0001) (Fig. 3B). Flow cytometric analysis of tumors from spleens of adult *Eµ-HDAC9* mice displayed a mature B-cell immunophenotypic profile (B220^dull^, IgD^low/neg^, IgM^high^, CD23^neg^ and CD21^neg^) (Fig. S4). *Eµ-HDAC9* B-cell tumors were primarily of splenic origin (Fig. 3C,D), with or without nodal involvement, expressed B220, indicating B-cell derivation, and were histologically defined as LPD, SMZL and DLBCL (Table 1). An additional 20% of *Eµ-HDAC9* mice either developed splenic B-cell lymphoma/leukemia (SBCL/L) or another hematological malignancy, such as histiocytic sarcoma (Table 1).

**Fig. 4.**
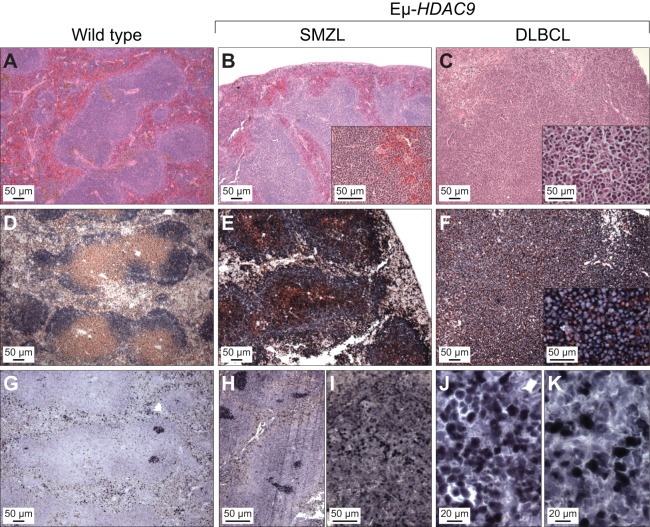
**Histopathological analysis of mature B-cell lymphomas in *Eµ-HDAC9* mice.** Representative images showing disorganization of lymphoid tissue and expression of B220, CD3 and BCL6. Mouse spleen sections from wild type, splenic marginal zone lymphoma (SMZL) and diffuse large B-cell lymphoma (DLBCL) cases (as indicated) were stained with hematoxylin-eosin (A-C), doubled stained with B220 (blue)/CD3 (brown) (D-F), or BCL6 (blue) (G-J). IRF4 expression is shown in panels I and K.

Molecular analysis of the rearranged IgH genes from *Eµ-HDAC9* B-NHL samples confirmed their monoclonal origin (Fig. 3E) with evidence of somatic hypermutation (SHM) of IgV genes in 4/7 (57%) of tumors (Fig. S5). Thus, we concluded that *Eµ-HDAC9* lymphomas and LPDs were derived from B-cell precursors, with evidence of transit through the GC or having experienced the GC reaction. In mice, the lymphomas and LPDs exhibited both GC and post-/non-GC immunophenotypes (Table 1). Immunohistochemical analysis revealed heterogeneous expression of BCL6, including cases that were below the detection level by immunohistochemistry (IHC), as previously observed in *Iμ-BCL6*-derived lymphomas (Cattoretti et al., 2005). *Eµ-HDAC9* tumors were also found to express variable levels of IRF4 (MUM1), a marker for GC B cells and plasma cells, and typically found in non-GC-type DLBCL (Fig. 4 and Table 1) (Falini et al., 2000).

**Fig. 5.**
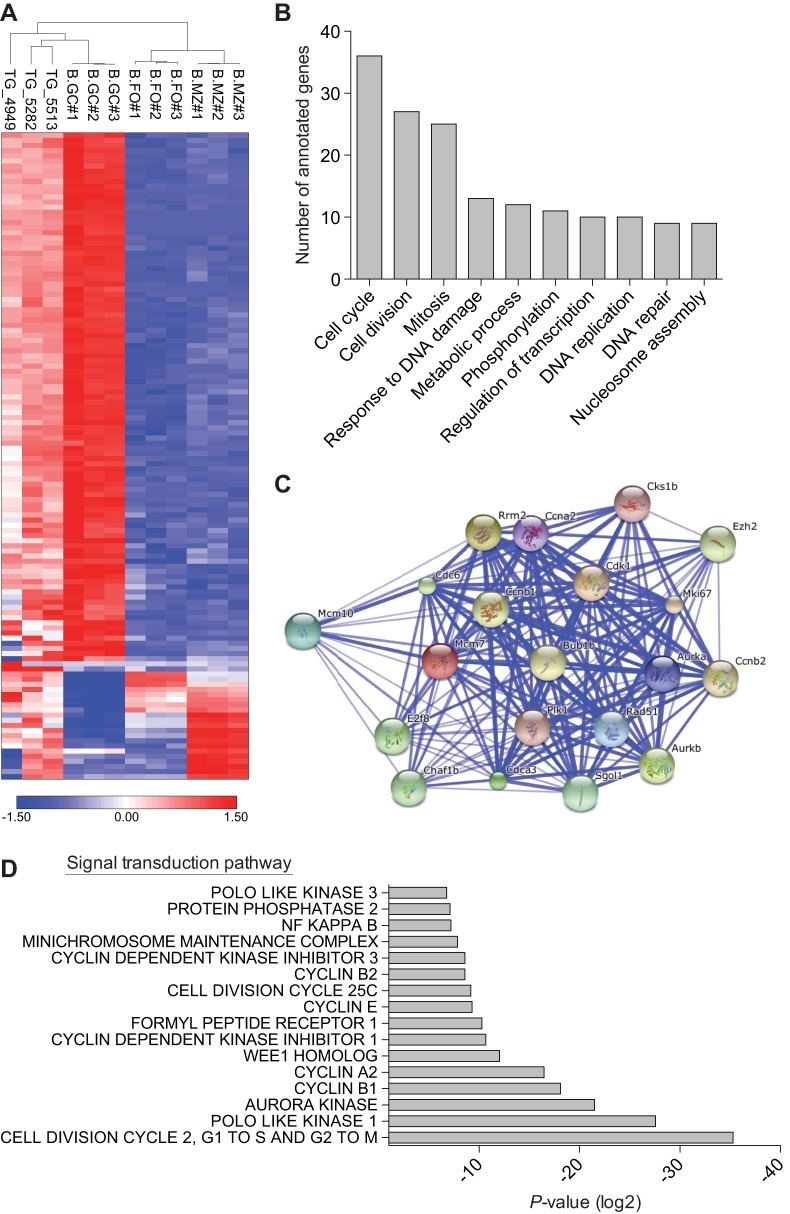
**Expression microarray analysis of *Eµ-HDAC9* tumors.** (A) Unsupervised hierarchical clustering of gene expression data from representative *Eµ-HDAC9-*derived (TG) lymphomas (*n*=3) versus normal murine mature B-cell subpopulations, including germinal center (GC), follicular (FO) and marginal zone (MZ) B cells (GSE15907). Color scale represents the range in relative expression changes (Z score) across samples, normalized by the standard deviation after log2 transformation (0, mean expression level; red, high expression; blue, low expression). (B) Functional annotation of upregulated gene expression in *HDAC9^TG^* tumors according to biological process. Values represent the number of upregulated genes for a given gene ontology (GO) term. A complete list of annotated genes for identified GO functional categories is shown in Table S5. Gene annotation was performed using Partek Genomics Suite 6.6. (C) Interactome of upregulated genes in *HDAC9^TG^* B-cell tumors. Interactions were analyzed using STRING v9.1. Thicker lines define stronger associations in the interactome. (D) Functional annotation of expression of signal transduction pathway genes upregulated in *HDAC9^TG^* tumors. Gene sets from *HDAC9^TG^* B-cell tumors were analyzed using GGA (Genomatix Genome Analyzer) and ranked according to *P*-value.

**Fig. 6.**
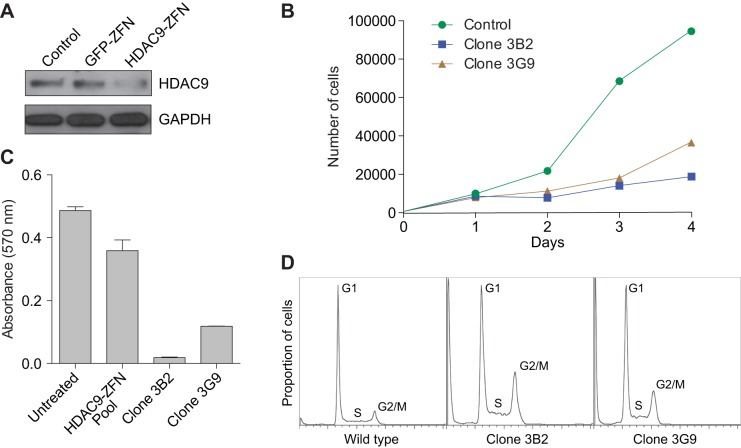
**HDAC9 regulates cell cycle progression.** (A) Immunoblot analysis of HDAC9 expression in untreated wild-type Raji B-cell non-Hodgkin lymphoma (B-NHL) cells (control, lane 1), and the mutants *GFP* ZFN-derived control (lane 2) and *HDAC9* ZFN-derived cells prior to single cell sorting (lane 3). Immunoblot analysis of GAPDH expression was used as loading control. (B) Cell growth curve of wild-type Raji control and representative ZFN-generated *HDAC9* mutant clones 3B2 and 3G9 obtained by single-cell sorting. (C) 3-(4,5-dimethylthiazol-2-yl)-2,5-diphenyltetrazolium bromide (MTT) assay of proliferation of *HDAC9* ZFN-derived clones. Shown are values for *HDAC9* ZFN-derived mutant clones 3B2 and 3G9, a pool of unsorted *HDAC9* ZFN mutants, and wild-type cells and untreated Raji cells. (D) Effects of HDAC9 depletion on cell cycle progression are depicted in histogram plots generated by propidium-iodide uptake.

We next performed gene expression analysis, and a comparison of three representative DLBCLs from *Eμ-HDAC9* mice versus normal B-cell populations indicated that *Eµ-HDAC9* B-cell tumors cluster with GC B cells and separately from non-GC B cells, further supporting a GC origin, comparable with human tumors (Fig. 5A). We also compared the expression pattern of DLBCLs from *Eμ-HDAC9* mice with three age- and gender-matched wild-type controls, identifying a total of 1469 upregulated and 307 downregulated transcripts (Table S2). Among the upregulated genes, pathway analysis using KEGG and gene ontology (GO) annotated databases revealed enrichment for genes involved in the cell cycle, cell division and response to DNA damage (Fig. 5B, Table 2, and Tables S3 and S4). Pathway-based hierarchical clustering (Good, 2000) for genes differentially expressed in *Eμ-HDAC9* mice (Tables S5, S6 and S7) confirmed regulation of cell-cycle-related genes and pointed to modulation of MAPK/ERK pathways. Furthermore, analysis of predicted protein–protein interactions between KEGG-classified genes from *HDAC9^TG^* B-cell tumors (Table S2) showed high network connectivity (Fig. 5C). A number of differentially expressed genes from the *Eμ-HDAC9*-driven B-cell tumors were identified as direct targets for BCL6 and p53 that have roles in apoptosis, the cell cycle and B-cell receptor (BCR) signaling (Table S2). Additionally, signal transduction pathways revealed substantial enrichment for genes involved in G1/S and G2/M transition followed by the Polo-like kinase 1 (PLK1) pathway (Fig. 5D). Among the most upregulated genes found in *HDAC9^TG^* B-cell tumors were those encoding factors such as Plk1, Birc5, Cdk1, Aurka, Aurkb and Chek1, which are involved in proliferation and survival, G1/S and G2/M transitions, mitosis and DNA-repair/checkpoint-mediated arrest (Table 2). A role for HDAC9 in proliferation and control of the cell cycle was confirmed by zinc-finger nuclease (ZFN)-mediated gene editing of *HDAC9* (Fig. 6). Monoallelic knockout of *HDAC9* in the Raji Burkitt's lymphoma cell line (biallelic forms were non-viable; data not shown) led to reductions in HDAC9, consistent with a decrease in gene dosage (Fig. 6A). This resulted in growth inhibition (Fig. 6B,C) and an increase in the proportion of cells in S and G2/M phases (Fig. 6D).

**Table 2.**
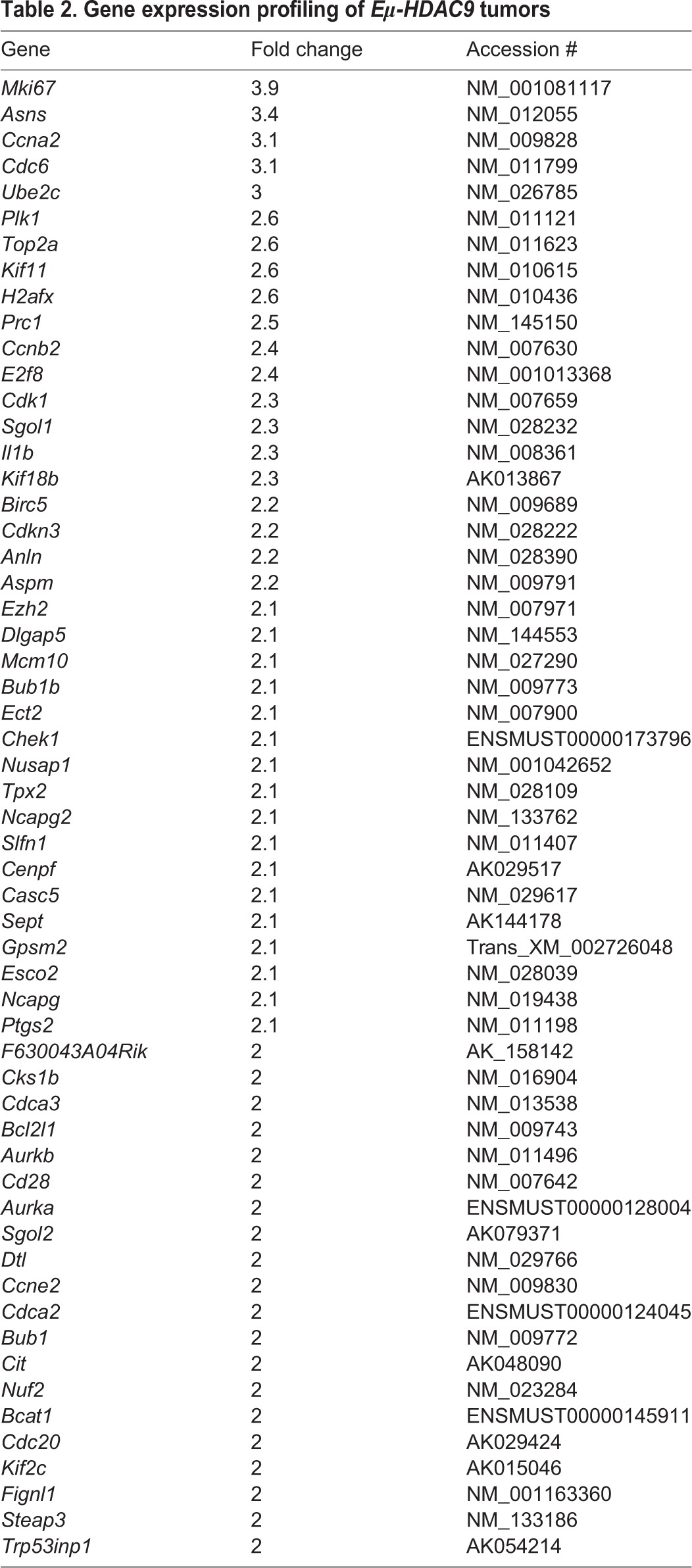
**Gene expression profiling of *Eμ-HDAC9* tumors**

**Fig. 7.**
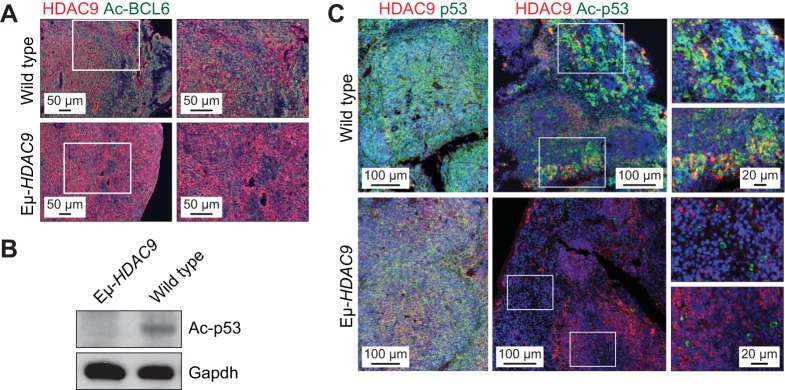
***Eµ-HDAC9* tumors display deregulated acetylation of BCL6 and p53.** (A) IHC triple-immunostaining using the ABC-TSA method in mouse spleens from *Eμ-HDAC9* and wild-type tumors, showing HDAC9 (red) expression in conjunction with levels of acetylated (Ac)-BCL6 (green). (B) Immunoblot analysis of Ac-p53 in *Eµ-HDAC9* and wild-type spleen. GAPDH was used as a loading control. (C) ABC-TSA immunofluorescence analysis of Ac-p53 (green) and HDAC9 (red) in spleen in *Eμ-HDAC9* and wild-type controls.

A potential role for HDAC9 in the pathogenesis of B-NHL is strengthened by its interaction with BCL6 (Petrie et al., 2003), a transcriptional repressor crucial for GC formation (Basso and Dalla-Favera, 2012) and whose deregulated expression in B cells leads to LPD and B-NHL (Cattoretti et al., 2005). BCL6 directly recruits class-II HDACs through its zinc-finger domain (Lemercier et al., 2002) and its transcriptional targets in GC B cells include *TP53*, thus modulating DNA-damage-induced apoptotic responses (Phan and Dalla-Favera, 2004). BCL6 and p53 function in a negative-feedback loop whereby p53 promotes *BCL6* expression, which in turn suppresses the expression of *TP53* (Margalit et al., 2006; Phan and Dalla-Favera, 2004). The BCL6–p53 axis is further modulated by post-translational acetylation of BCL6, which leads to its inactivation. Indicative of a potential role for HDAC9 in the acetylation of BCL6 *in vivo*, acetylated BCL6 was found to be abundant in normal mouse spleens but undetectable in *HDAC9^TG^* tumors (Fig. 7A). p53 is also post-translationally modified by acetylation, which is indispensable for its transcriptional activity in response to DNA damage and stress (Tang et al., 2008). Highlighting the loss of p53 tumor suppressor function as a contributory factor in the development of B-cell lymphomas, B-cell-specific disruption of *TP53* leads to the development of B-NHL (Chiang et al., 2012). Consistent with this, as well as with recent research demonstrating that HDAC9 binds to the *TP53* promoter to repress gene expression (Zhao et al., 2015), we found diminished levels of total p53 as well as Lys379 acetyl-p53 in *Eµ-HDAC9* tumors (Fig. 7B,C).

## DISCUSSION

*HDAC9* is highly expressed in B-NHL cell lines and patient samples (Petrie et al., 2003; Sun et al., 2011), and its locus, chromosome 7p21.1, is frequently amplified in B-NHL (Bea et al., 2005; Bentz et al., 1999; Monni et al., 1996; Rubio-Moscardo et al., 2005; Tagawa et al., 2005). In order to establish whether deregulated expression of HDAC9 in the lymphoid compartment could generate a disease phenotype, we designed a GEM model in which a human *HDAC9* transgene was expressed under the control of the *Eµ* promoter. Our hypothesis was based on evidence of deregulation of HDAC9 expression in B-NHL cell lines and patient samples. The occurrence of B-NHL in these transgenic mice strongly indicates a link between deregulated HDAC9 expression and lymphoid neoplasia, the first time that overexpression of a histone deacetylase in mice has resulted in a cancer phenotype. This is also, to our knowledge, the first time that expression of a single epigenetic transgene has led to lymphoma – expression of mutated polycomb-group gene *EZH2* fails to drive lymphomagenesis unless in a background of overexpressed *BCL2* or *Myc* (Béguelin et al., 2013; Berg et al., 2014). This does not, however, rule out a requirement for the acquisition of additional mutations in order for progression to lymphoma in *Eµ-HDAC9* mice. Our results are also in agreement with a recent study utilizing an *MRL/lpr* GEM model of systemic lupus erythematosus with *HDAC9* deficiency (Yan et al., 2011). Here, *MRL/lpr* transgenic mice lacking *HDAC9* displayed decreased lymphoproliferation and expression of BCL6. Although the SMZL cases studied in *Eµ-HDAC9* mice were BCL6-negative, it is generally accepted that MZL in humans is derived from a post-GC B cell, with associated somatic mutations in the IgV_H_ gene (Dunn-Walters et al., 1998; Miranda et al., 1999; Zhu et al., 1995). Moreover, progression of indolent lymphoma (SMZL) to a more aggressive lymphoma (DLBCL) is a frequent occurrence for many subtypes of indolent B-cell lymphomas and could explain the late onset of aggressive lymphomas observed in our transgenic model (Camacho et al., 2001; Freedman, 2005). The development of SMZL in *Eµ-HDAC9* mice is also consistent with results obtained in mice in which *TP53* was disrupted specifically in B cells, leading to the development of highly penetrant SMZL (Chiang et al., 2012).

Our results indicated that aberrant expression of HDAC9 in B cells leads to the upregulation of pathways that promote cell growth and survival, as well as impacting the activity and expression of key factors in lymphoma BCL6 and p53. The notion that transgenic expression of HDAC9 can promote lymphomagenesis, in part through deregulation of the activities of p53, is strengthened by studies suggesting that p53 might directly bind to the *HDAC9* promoter and repress its expression (Akdemir et al., 2014; Wei et al., 2006). Of note, the p53-binding site in the *HDAC9* promoter overlaps with a myocyte enhancer factor 2 (MEF2)-binding site, which, when bound by MEF2 family members, activates *HDAC9* gene expression (Haberland et al., 2007). Activating mutations of *MEF2B* (which occur in 11% of DLBCL and 12% of FL) have been reported to directly upregulate expression of BCL6 (the promoter of which also contains a MEF2-binding site) in GC B cells and drives DLBCL proliferation (Ying et al., 2013). It remains to be established whether mutant MEF2B can drive HDAC9 expression in B-cell lymphomas, but recent research has identified a novel MEF2D–BCL9 fusion protein associated with high-risk acute B-cell precursor lymphoblastic leukemia (ALL) that directly upregulates HDAC9 (Suzuki et al., 2016). High expression of HDAC9 has been independently linked to poor prognosis in ALL (Moreno et al., 2010).

In recent years, numerous structurally diverse HDAC inhibitors (HDACi) have emerged as clinical candidate therapeutic agents (West and Johnstone, 2014), including the recent development of class-IIa-specific HDACi (Lobera et al., 2013). Even in the case of cutaneous T-cell lymphoma (CTCL) where HDACi have shown efficacy as single-agent targeted therapies and been approved for use in the clinic (Prince and Dickinson, 2012), the use of HDACi in rational combinations will likely maximize their therapeutic potential. It is, therefore, of interest that genes upregulated in *Eµ-HDAC9* tumors, such as *Cdk1*, *Chek1*, *Aurka* and *Aurkb*, represent important clinical targets for which late-phase clinical trials are ongoing (Garrett and Collins, 2011; Lapenna and Giordano, 2009; Micel et al., 2013). The upregulation of Plk1 is also of potential clinical interest given that its high expression is a negative prognostic indicator in B-NHL (Liu et al,. 2007; Xu et al., 2013; Yim et al., 2013). PLK1 plays a crucial role at checkpoint controls during G2/M transition of the mitotic cell cycle (Barr et al., 2004) and inhibits p53 function directly by phosphorylation (Ando et al., 2004). Therefore, the *Eµ-HDAC9* GEM model could serve as a valuable tool both to better understand the molecular mechanisms involved in lymphomagenesis in humans and facilitate preclinical studies of new drugs and combination therapies.

## MATERIALS AND METHODS

### Generation of *Eμ-HDAC9* transgenic mice

FLAG-epitope-tagged full-length human *HDAC9* cDNA was cloned into the pEμSR vector, placing the *HDAC9*-sequence-containing oligonucleotide cassette downstream of the immunoglobulin heavy chain (IgH) enhancer (*Eμ*) and the *SRα* potent promoter (Bodrug et al., 1994). The *Eμ-HDAC9* transgenic fragment was isolated from the vector by enzymatic digestion using the *Not*I restriction sites and injected into B6CBAF1 pronuclei. Mice were backcrossed and maintained in a C57BL/6 background to generate three transgenic lines: 1468, 1469 and 1839. PCR genotyping was performed using SV40 primers: F: 5′-GGAACTGATGAATGGGAGCA-3′ and R: 5′-GCAGTGCAGCTTTTTCCTTT-3′. Mice were housed and maintained in accordance with UK Home Office regulations. Animals were monitored and analyzed from birth to 23 months of age and sacrificed if showing signs of illness. Statistical analysis was performed using Prism (GraphPad Software). Kaplan–Meier cumulative survival and the log-rank (Mantel–Cox) test were used to determine tumor-free survival and the χ^2^ test was used to compare B-NHL incidence in *Eµ-HDAC9* mice versus wild-type controls. *P*<0.05 was considered statistically significant. All experimental protocols were monitored and approved by The Institute of Cancer Research Animal Welfare and Ethical Review Body, in compliance with guidelines specified by the UK Home Office Animals (Scientific Procedures) Act 1986 and the United Kingdom National Cancer Research Institute guidelines for the welfare of animals in cancer research (Workman et al., 2010). ARRIVE guidelines were applied when reporting *in vivo* experiments (Kilkenny et al., 2010).

### Ig gene rearrangements analysis

Genomic DNA was isolated from tumor specimens and prepared using All Prep Kit (Qiagen). Primers for detection of Ig rearrangements were described previously (Ehlich et al., 1994). D-J_H_ rearrangements of the heavy chain (J_H_) locus were amplified and detected in a multiplex PCR reaction using two upstream primers, DFL/DSP and DQ52, together with one reverse primer positioned downstream of J_H_4 (Mårtensson et al., 1997). For mutational analysis, the rearranged Ig variable heavy chain genes were amplified from genomic DNA as previously described (Cattoretti et al., 2005). V-DJ_H_ rearrangements were analyzed in separate PCR reactions using primers V_H_J558a (5′-CAGGTCCAGCTGCAGCAGTCTGG-3′), V_H_7183b (5′-GTGAAGCCTGGAGGGTCCC-3′), V_H_Q52 (5′-CAGGTGCAGCTGAAACAGTCA-3′) and reverse J_H_4 primer (5′-TGAGGAGACGGTGACTGAGGTTCC-3′). The primers amplified all rearrangement products between J_H_4 and V_H_J558, V_H_7183 or V_H_Q52 where four different bands would be expected by the combination of each primer set. PCR cycling conditions were: 95°C for 5 min, 95°C for 30 s, 63°C for 30 s and 72°C for 2 min, for 35 cycles. PCR products were gel-purified using the QIAQuick method (Qiagen) and directly sequenced using the ABI PRISM 3130*xl* Genetic Analyzer (Applied Biosystems). Sequences were aligned to those in the international ImMunoGeneTics information system (IMGT) database.

### Flow cytometry and cell sorting

Single-cell suspensions were obtained from dissected tissues, washed in phosphate buffered saline (PBS) supplemented with 0.2% FBS, filtered through a 45 µm cell strainer and red blood cells were lysed using ammonium chloride solution (STEMCELL Technologies). The antibody combination used for tumor analysis included: CD23, CD19, IgM, IgD, CD21 and B220. Anti-mouse conjugated antibodies were obtained from eBioscience and BioLegend. Cell sorting of splenic CD19-positive mouse B cells was carried out using immunomagnetic isolation with CD19-labeled beads (EasySep Mouse CD19 Positive Selection Kit, STEMCELL Technologies) and purity assessed using anti-mouse PE-conjugated CD19 antibody (eBioscience). Data were acquired on a FACS LSRII analyzer (BD Biosciences) and analyzed using FlowJo software (Tree Star).

### Transgenic transcript detection

Total RNA from mouse bone marrow and spleen was extracted using TRIzol reagent (15596-026, Ambion) following the manufacturer's instructions. Reverse transcription was performed following standard protocol by using AccessQuick Master Mix (A1701, Promega). Real-time quantitative PCR was carried out using Fast SYBR Green Master Mix (4385612, Applied Biosystems). Primer sets used to detect mouse and human *HDAC9* transcripts in B-cell subsets: F: 5′-TCTGGATGTTCACCATGGAA-3′ and R: 5′-CACTGCCAGGGAAAAAGTTC-3′. Primer sets to detect mouse *Hdac9*: F: 5′-GGTGATGATTCTCGGAAATTCT-3′ and R: 5′-GAAGCCAGCTCAATGACACA-3′. *Abl1* mRNA expression was used as normalization control, F: 5′-CAGCGGCCAGTAGCATCTGACTT-3′ and R: 5′-GCTTCACACCATTCCCCATT-3′.

### Purification of B-cell subsets

Single-cell suspensions from spleen (*n*=3) and bone marrow (*n*=5) from 8- to 12-week-old mice (transgenic and wild type) were prepared as previously described (Green et al., 2011). Bone marrow and spleen B-cell subpopulations were identified and sorted by three-color FACS method (Green et al., 2011). Anti-mouse antibodies CD43, IgM and B220 were used to obtain Pro-B, Pre-B and Mature-B-cell fractions from bone marrow. Splenic B-cell subtypes, follicular and marginal zone, were purified using anti-mouse CD19, CD21 and CD23 antibodies, whereas a combination of anti-mouse B220, IgM and CD93 was used to sort for transitional B cells. Each subset was above 90% purity. Purified cells were re-suspended in TRIzol reagent (Life Technologies) for further RNA extraction.

### Histopathology and immunohistochemistry (IHC)

Tissues were freshly collected and fixed in 10% formalin for 24 h, embedded in paraffin and sectioned. Following deparaffinization and rehydration, samples were pre-treated with antigen-unmasking solution (Vector Laboratories, H-3300). After pre-treatment, tissue sections were blocked in Tris-NaCl buffer, washed in PBS and permeabilized in 0.5% Triton X-100. Slides were washed in Tris-NaCl-Tween buffer and reacted with primary antibody overnight at 4°C. Preliminary experiments were performed to determine optimal dilutions for each primary antibody used. Detection methods included: standard IHC, Vectastain Elite ABC system, Vector Laboratories or EnVision system (Dako) DAB staining and ABC-tyramide signal amplification (TSA Plus Fluorescence System, Perkin Elmer) (see below). Histopathological evaluation of *HDAC9^TG^* tumors was performed using hematoxylin and eosin (H&E)-stained sections. Immunophenotypic characterization of the *HDAC9^TG^* lymphomas was performed using antibodies against B220, PAX5, CD3, BCL6 and IRF4 as previously described (Mandelbaum et al., 2010). Human HDAC9 expression was assessed by IHC using tissue microarrays and/or individual tumor sections of 59 B-NHLs and three CHLs using a specific antibody generated against the C-terminal region of HDAC9 (Petrie et al., 2003). The B-NHL group consisted of 34 DLBCLs, nine FLs, five MZLs, six MCLs and two chronic lymphocytic leukemias/small lymphocytic lymphomas (CLLs/SLLs). Nuclear expression intensity of lymphomas was compared with that of rectal adenocarcinoma cells (positive control). If equal or higher intensity than control, HDAC9 expression was considered high (score 2+), and, if low, scored 1+.

### Immunoblotting and antibody validation

Cell lysates were prepared using RIPA buffer supplemented with protease inhibitors (Roche). Rat monoclonal (IgM) antibody specific to the C-terminus of human HDAC9 (clone 45a7b5b) was developed using a synthetic peptide (DVEQPFAQEDSRTAG) conjugated to Diphtheria toxoid (Mimotopes), corresponding to unique amino acids 1046-1060. Validation was performed using Mini-PROTEAN II Multiscreen Apparatus (Bio-Rad). Other antibodies used for immunoblots included mouse monoclonal anti-FLAG M2 antibody (F1804, Sigma), β-actin loading-control antibody (BA3R) (MA5-15739, Thermo Scientific) and acetyl-p53 (Lys379) (PA5-17287, Thermo Scientific).

### IHC and immunofluorescence detection methods

#### Single immunolabeling using ABC-TSA

Formalin-fixed paraffin-embedded tissue sections were dried for 45 min at 58°C, followed by deparaffinization and hydration in Histoclear (National Diagnostics) and a graded series of ethanol, respectively. Samples were pretreated by microwave incubation in a pH 6.0 citrate-based antigen-unmasking solution (Vector Laboratories, H-3300) followed by 2×5 min washes in PBS and permeabilization in 0.5% Triton X-100 (in PBS) for 20 min at room temperature (RT). Samples were washed in PBS prior to blocking for 30 min at RT in Tris-NaCl (TNB) blocking buffer with subsequent incubation in the monoclonal anti-HDAC9 antibody (clone 45a7b5b, 1/100 dilution) overnight at 4°C. Primary antibody was followed by biotinylated rabbit anti-rat secondary antibody for 30 min at RT. Endogenous peroxidase activity was inactivated by incubation with 3% hydrogen peroxide in methanol for 15 min at RT. After washing in Tris–NaCl–Tween-20 (TNT) buffer, samples were incubated in streptavidin (SA)-horseradish peroxidase (HRP) (Vectastain ABC Elite Kit, Vector Laboratories, PK-6104) for 45 min at RT. Following this, FITC fluorophore tyramide (Perkin Elmer, NEL741) was added for 10 min at RT, which results in the deposition of numerous fluorophore labels adjacent to the HRP. This fluorescent signal was then converted to a chromogenic signal by the addition of HRP-labeled anti-FITC (Perkin Elmer, NEF710), 1/200 dilution in TNB for 45 min at RT, 3×5 min washes in TNT and incubation in chromogen 3-amino-9-ethylcarbazole (AEC) (BUF019B, AbD Serotec). Hematoxylin QS (Vector Laboratories, H-3404) was utilized for nuclear counterstaining and the sections mounted in Vectamount aqueous mounting medium (Vector Laboratories, H-5501). Images were collected on a Zeiss Axioskop 2 light microscope utilizing Axiovision 4.4 software.

#### Double/triple labeling with ABC-TSA

The following immunolabeling reactions were applied using ABC-TSA method as described elsewhere (Asson-Batres and Smith, 2006). Preliminary controls showed no antibody carryover when one primary antibody was a rabbit polyclonal and the other was a rat monoclonal antibody, or when both primary antibodies were made in rabbits. Images were collected on Zeiss LSM700 confocal microscope using Zen 2009 software with sequential collection to prevent any spectral crosstalk.

##### Double immunolabeling

Formalin-fixed paraffin-embedded tissue sections were dried for 45 min at 58°C followed by deparaffinization and hydration in Histoclear (National Diagnostics) and a graded series of ethanol, respectively. Samples were pre-treated by microwave incubation in a pH 6.0 citrate-based antigen-unmasking solution (Vector Laboratories, H-3300) followed by 2×5 min washes in PBS and permeabilization in 0.5% Triton X-100 in PBS for 20 min at RT. Samples were then washed in PBS prior to blocking for 30 min at RT in TNB blocking buffer with subsequent incubation in the monoclonal anti-HDAC9 antibody (clone 45a7b5b, 1/100 dilution) overnight at 4°C. Primary antibody was followed by biotinylated rabbit anti-rat secondary antibody (Vectastain ABC Elite Kit, Vector Laboratories, PK-6104) for 30 min at RT. Endogenous peroxidase activity was quenched by incubation in 3% hydrogen peroxide (in methanol) for 15 min at RT. After washing in TNT buffer, samples were incubated in SA-HRP (Vectastain, Vector Laboratories ABC Elite Kit, PK-6104) for 45 min at RT. Next, a Cy3 fluorophore tyramide (Perkin Elmer, NEL741) was added (1/50 dilution prepared in the supplied amplification buffer) for 10 min at RT, which results in the deposition of numerous fluorophore labels adjacent to the HRP. Slides were washed 3×5 min in TNT buffer, any remaining HRP was deactivated by incubating in 3% hydrogen peroxide (in methanol) for 15 min at RT. A second primary antibody was added sequentially [anti-acetylated p53 (Lys379) Thermo Scientific, cat. # 17287, 1/100 dilution] and the protocol was repeated with the FITC fluorophore tyramide (Perkin Elmer, NEL741) utilized to detect acetylated p53. Slides were counterstained with To-pro 3 iodide (Life Technologies, T-3605) and mounted in Vectashield (Vector Laboratories, cat. # H-1000) mounting medium. Negative control slides were prepared by excluding the primary antibody, by excluding the conjugated secondary antibody-fluorophore and by excluding TSA reagents; negative controls showed no immunoreactivity. Single-labeling experiments carried out to observe the patterns of staining of each primary antibody validated the double-immunostaining results.

##### Triple immunolabeling

First, the primary antibodies monoclonal rat anti-HDAC9 (clone 7b5b, 1/100 dilution) and rabbit polyclonal anti-acetylated lysine (Millipore 06-933, 1/500 dilution) were added together and left overnight at 4°C. Following this, secondary anti-rat biotinylated antibody (1/200 dilution) was added and the TSA protocol was carried out using Cy3 fluorophore tyramide (Perkin Elmer, NEL753). Next, HRP was inactivated before the secondary antibody against acetylated lysine was added (anti-rabbit biotinylated) and the TSA protocol followed using FITC fluorophore tyramide (Perkin Elmer, NEL753). The HRP was once again inactivated with 3% hydrogen peroxide (in methanol) for 15 min at RT and rabbit polyclonal anti-BCL6 antibody (ab19011) (1/1000 dilution) was added (overnight at 4°C). Following this, the TSA protocol was applied using Cy5 fluorophore tyramide (Perkin Elmer, NEL745). Secondary-antibody controls were performed to detect any non-specific background staining. Single-staining controls were carried out as in the same-species double-labeling experiments.

#### IHC of human tumors

The EnVision detection system HRP/DAB+ (Dako) was used as previously described (Kim et al., 2009). Briefly, tissue sections were deparaffinized in xylene and rehydrated in ethanol following treatment in pre-heated target retrieval solution. Following washes, serum-free blocking solution was applied for 40 min at RT. In-house anti-HDAC9 monoclonal antibody was used overnight at 4°C then treated with polymer/HRP and DAB. After washes, the slides were counterstained with hematoxylin, dried and mounted with Permount. Photomicrographs were captured using an Olympus BX41 dual head light microscope equipped with an Olympus Q-Color 5 digital camera (Olympus America).

#### Antibodies IHC/IF

Primary antibodies included polyclonal rabbit anti-Ac-p53 (Lys379) (Thermo Scientific), monoclonal rat anti-HDAC9 antibody (clone 45a7b5b), monoclonal mouse anti-FLAG M2 (Sigma), polyclonal rabbit anti-acetylated lysine (Millipore 06-933), polyclonal rabbit anti-BCL6 (ab19011), anti-CD45R(B220) (ab64100) and anti-CD3 (ab5690). Secondary antibodies included: biotinylated rabbit anti-rat secondary antibody (Vector Laboratories, PK-6104), biotinylated horse anti-mouse secondary (Vector Laboratories, PK6102) and biotinylated goat anti-rabbit secondary (Vector Laboratories, PK6101). Fluorochromes and chromogens included SA-HRP (Vector Laboratories, PK-6102), FITC fluorophore tyramide (Perkin Elmer, NEL741, NEL753), FITC-HRP (Perkin Elmer, NEF710), Cy3 fluorophore tyramide (Perkin Elmer, NEL741, NEL753), Cy5 fluorophore tyramide (Perkin Elmer, NEL745) and AEC (AbD Serotec, BUF019B).

### High-density SNP array analysis

Genome-wide DNA profiles were obtained from high-molecular-weight genomic DNA of DLBCL patients using the Affymetrix Genome-Wide Human SNP Array 6.0 (Affymetrix) following the manufacturer's instructions. Image data analysis and quality control for the hybridized samples were performed using the Affymetrix Genotyping Console 3.0.1 software, and only samples passing the Affymetrix recommended contrast QC and SNP call rates threshold (in the Birdseed v2.0 algorithm) were considered for analysis. Affymetrix CEL files and corresponding SNP genotype call files generated by the Affymetrix Genotyping Console tool were then analyzed using the dCHIP software. Model-based expression was performed using the perfect-match/mismatch (PM/MM) model to summarize signal intensities for each probe set. Probe intensity data for each array were normalized using a diploid reference set of three normal (non-tumor) DNA samples that had been processed and hybridized in the same experiment as the tumor samples. The standard invariant-set normalization approach in dCHIP was implemented by a karyotype-guided normalization method as previously described (Mullighan et al., 2007; Pounds et al., 2009). To identify regions of amplification and deletion, the circular binary segmentation algorithm was applied to the SNP array data as described (Mullighan et al., 2007). The following criteria were used to obtain candidate genomic regions (gains or loss): (1) mean log2 ratios of ≥0.2 or ≤−0.2; (2) ≥8 SNP markers within a segment. The results of the CBS algorithm were then compared to those of dCHIP. To exclude calls of genomic gains or loss arising from inherited genomic copy number variants (CNVs), the dCHIPSNP algorithm was also applied to 130 normal DNAs from an independent study as well as to 230 normal DNAs from the HapMap project; alterations identified in the pool of reference samples were excluded. In addition, CNVs were excluded if present in the Database of Genomic Variants (http://projects.tcag.ca/cgi-bin/variation/gbrowse/hg18/).

### Gene expression analysis

Affymetrix GeneChip Mouse Gene 1.0 ST hybridizations were performed using biotin end-labeled cDNA prepared from CD19-positive B cells isolated from tumors. Unsupervised hierarchical clustering was performed on gene expression data from representative *HDAC9^TG^*-derived lymphomas versus normal murine mature B-cell subpopulations, including GC and non-GC (follicular and marginal zone) B cells (GeneChip Mouse Gene 1.0 ST Arrays). Data from normal B-cell subsets were obtained from the Immunological Genome Project (GSE15907) (www.immgen.org). Only probes with minimal expression level equal to 10 and minimal standard deviation of 1.5 (log2 transformed) were considered. The hierarchical clustering algorithm is based on the average-linkage, Pearson correlation.

Gene data sets were also analyzed for interactions and pathways using: GGA (Genomatix Genome Analyzer, https://mygga.genomatix.de), the Search Tool for the Retrieval of Interacting Genes/Proteins STRING v9.1 (http://string-db.org) to develop interactomes or networks (Franceschini et al., 2013) and the KEGG pathway database (Kanehisa et al., 2004). GO (Ashburner et al., 2000) clustering was performed with csbl.go (Ovaska et al., 2008). Partek Genomics Suite 6.6 was additionally used for data set analysis and comparisons.

### Zinc-finger nuclease (ZFN) knockout of HDAC9

ZFNs targeting human *HDAC9* sequence were obtained from Sigma-Aldrich (CompoZr Knockout Zinc Finger Nucleases, CKOZFND9935-1KT). The ZFN binding-cutting site (in lowercase) was 5′-CTCTGGTCCCAGTTCACCaaacaATGGGCCAACTGGAAGTG-3′. Delivery of ZFN was performed following the manufacturer's protocol (Sigma-Aldrich). The human Burkitt's lymphoma cell line Raji (ATCC CCL-86) was used for the study. Cells were maintained in RPMI medium 1640 (1×) (Gibco) supplemented with 10% FBS (Sigma-Aldrich) and grown in a 5% CO_2_ incubator at 37°C. Cells were transfected by nucleofection (electroporation) using Amaxa Cell Line Nucleofector Kit V (Lonza), and grown for 48 h followed by single-cell sorting in a 96-well format using BD FACS Aria (BD Biosciences). After 3-4 weeks, single-cell-derived clones were screened and analyzed using CEL-I assay (SURVEYOR mutation detection assay) following the manufacturer's instructions (Transgenomic). Genomic DNA was obtained by high-throughput HotSHOT DNA preparation method in 96-well plates. ZFN mutant clones were confirmed by sequencing.
